# Characterization of spatially mapped volumetric molecular ultrasound signals for predicting response to anti-vascular therapy

**DOI:** 10.1038/s41598-022-26273-0

**Published:** 2023-01-30

**Authors:** Cody A. Keller, Shaya Zarkesh, Jianhua Zhou, Amelie M. Lutz, Dimitre Hristov, Aya Kamaya, Ahmed El Kaffas

**Affiliations:** 1 Department of Radiology, School of Medicine, Stanford University, Stanford, CA USA; 2 Department of Radiation Oncology, Stanford University, Stanford, CA USA

**Keywords:** Cancer imaging, Tumour biomarkers, Tumour heterogeneity, Predictive markers

## Abstract

Quantitative three-dimensional molecular ultrasound is a promising technology for longitudinal imaging applications such as therapy monitoring; the risk profile is favorable compared to positron emission tomography and computed tomography. However, clinical translation of quantitative methods for this technology are limited in that they assume that tumor tissues are homogeneous, and often depend on contrast-destruction events that can produce unintended bioeffects. Here, we develop quantitative features (henceforth image features) that capture tumor spatial information, and that are extracted without contrast destruction. We compare these techniques with the contrast-destruction derived differential targeted enhancement parameter (dTE) in predicting response to therapy. We found thirty-three reproducible image features that predict response to antiangiogenic therapy, without the need for a contrast agent disruption pulse. Multiparametric analysis shows that several of these image features can differentiate treated versus control animals with comparable performance to post-destruction measurements, suggesting that these can potentially replace parameters such as the dTE. The highest performing pre-destruction image features showed strong linear correlations with conventional dTE parameters with less overall variance. Thus, our study suggests that image features obtained during the wash in of the molecular agent, pre-destruction, may replace conventional post-destruction image features or the dTE parameter.

## Introduction

Functional assessment of tumor response to cancer therapy is needed for individualized therapy. The Response Evaluation Criteria in Solid Tumors^1^ (RECIST 1.1) criteria is currently used as the gold standard for tumor response evaluation, despite two major limitations. First, assessment of response (measurable change) does not occur until 90 days after therapy initiation which is a long time for patients to endure toxic side effects especially if the treatment is not effective. Second, size change may not reflect response in cytostatic oncologic therapies.

Quantitative functional imaging has been shown to help address these issues in multiple imaging modalities^2–16^ by identifying clinical response to cytostatic anti-angiogenic therapies earlier than anatomic imaging. Three-dimensional molecular contrast-enhanced ultrasonography (3D-mCEUS) has shown promising efficacy and safety in preclinical animal^17–31^ and pilot human^32^ data. Treatment response may be predicted as early as 24 h after treatment initiation based on conventional quantitative parameters^33^. However, the main parameter commonly employed in most of these studies relies on the differential targeted enhancement (dTE), where 5 + minutes of cine image acquisition and ultrasonic destruction of microbubble contrast is used to infer the bound fraction of targeted microbubbles^34^. These destruction pulses have been shown to generate unintended bioeffects in animal models^35,36^ limiting translation to clinical study. Further, prior work developing quantitative methods for therapy monitoring, such as the dTE, produce parameters averaged over the tumor region of interest (ROI), which may be overlooking valuable spatial information toward predicting therapy response. Many studies utilizing analysis of the image features of tumor tissues^37–45^ offer support of exploration for more granular parameters of heterogeneities inherent to tumor tissues.

As such, using conventional statistical methods and multiparametric analysis, we assess the feasibility of replacing the dTE parameter with image features extracted from data without contrast destruction. These image features include second-order statistical properties of signal intensity distribution and are expected to better capture tissue heterogeneities of tumors^46^, which, in turn, may provide improved discrimination of therapy effects. Raw 4D cine-image data were preprocessed into static 3D intensity maps using intensity projection methods. Spatial maps of the dTE and standard deviation of pre- and post-destruction signal intensities were obtained through a sliding window method^16^. Image feature data were then stratified by the size of raw imaging dataset used and the need for a destruction pulse to generate eleven intensity maps. With this approach, we compare the treatment effect discrimination of image features extracted from image data with and without contrast destruction. Our results demonstrate a small set of image features extracted from intensity maps from 10 cine frames prior to contrast destruction that perform as well or better in classifying treated and control animals. Thus, those image features could be used to quantify molecular ultrasound signals without the need for potentially invasive destruction events while imaging.

## Results

### Features extracted from intensity projection maps are repeatable and statistically significant in detection of treatment response

**Figure 1:**
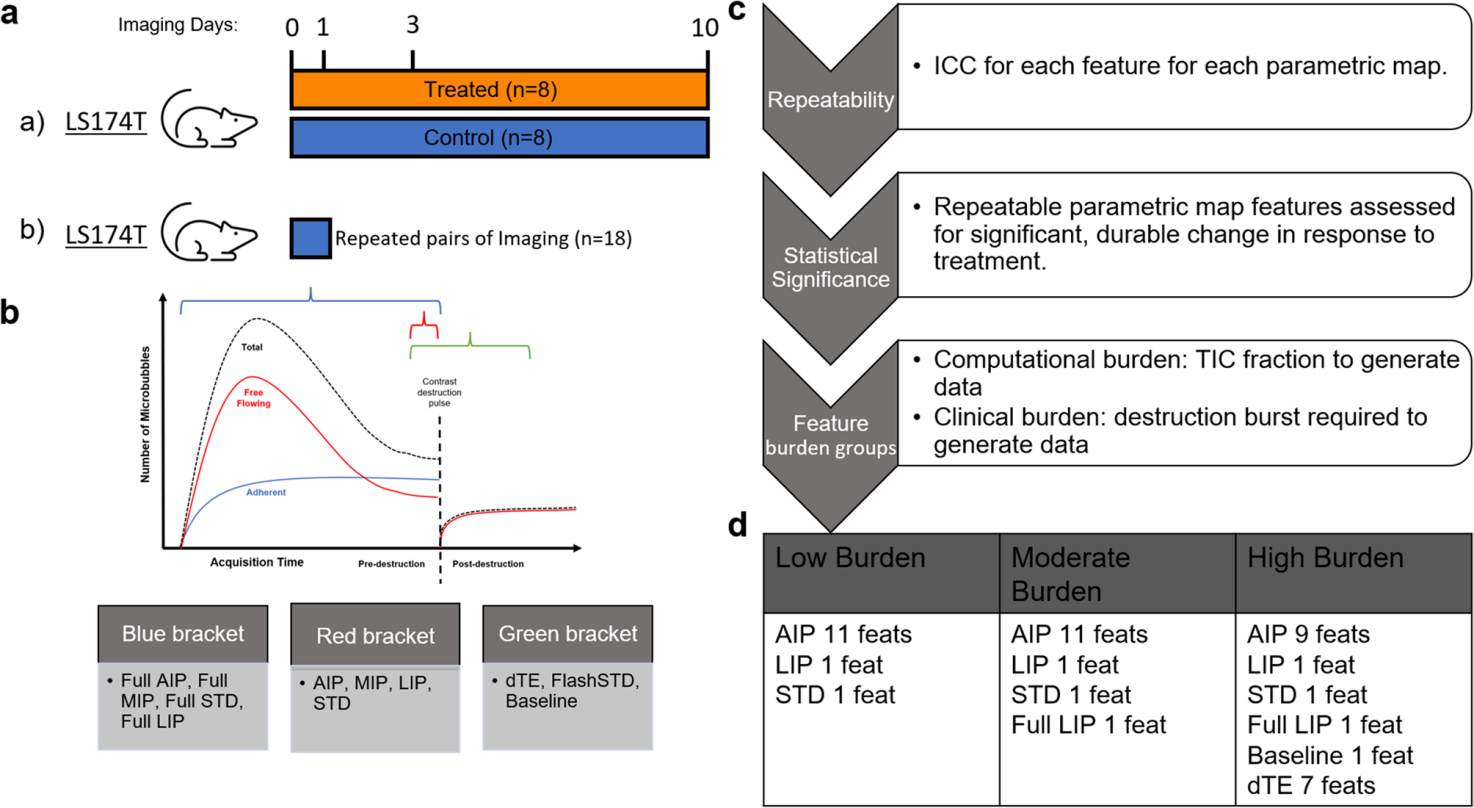
*AIP* Average intensity projection, *MIP* maximum intensity projection, *dTE* differential targeted enhancement, *STD* standard deviation, *LIP* lowest intensity projection, *ICC* intra-class correlation coefficient, *TIC* time-intensity curve. Overview of feature extraction and selection process. (**a**) Schematic summary of US imaging data acquisition and mouse cohorts. (**b**) Representative TIC for molecular imaging showing the fraction of acquired cine frames from which intensity maps are derived. (**c**) Stepwise statistical feature selection process leading to formation of feature sets stratified by burden of computational requirement and/or necessity of destruction US pulse. (**d**) Summary of image feature sets based on expected clinical burden for obtaining after statistical feature selection and correlation elimination.

Intensity maps derived from the four-dimensional (i.e. 3D volumes + time cine) imaging data represent a voxel-by-voxel transformation of assigned intensity values across the 3D volume of interest (VOI). Sampled properties of intensity dynamics for each voxel are taken throughout a predefined period of image acquisition (Fig. 1b). We generated 11 total intensity maps based on the uniform sampling of statistical properties (average, maximum, minimum, standard deviation, and dTE) of intensity values each voxel takes throughout a pre-defined period of total cine image acquisition. A complete list of these intensity maps with descriptions and representative image data are available in Supplementary Table 1 and Supplementary Fig. 1, respectively. From this, three categories of maps are apparent: (1) intensity properties sampled from all cine data prior to destruction, (2) intensity properties sampled from the final 10 frames of cine data prior to destruction, and (3) intensity maps including post-destruction data representing the dTE. This preprocessing step compresses the raw voxel intensity dynamics to a more computationally manageable 3D data set. Additionally, molecular signal heterogeneities within discrete statistical contexts are limited to a predefined portion of the total cine image acquisition. Thus, by uniformly sampling from properties of intensity value distribution, we generate datasets robust to noise introduced to raw data through inconsistencies in probe handling or contrast administration.

We carried out statistical analysis of 91 image features extracted from each of 11 intensity maps assessing for repeatability of measured image feature values with intraclass correlation coefficient (ICC; ICC > 0.80) and the detection of a significant, and durable change from baseline in response to treatment with Wilcoxon Signed-Rank test (WSR; p < 0.05), (Fig. 1c). Repeatability analysis with ICC was performed on serial 3D-mCEUS images taken from a separate cohort of mice (Fig. 1a), testing repeatability of each image feature for each intensity map. 54 of the 91 image features tested showed sufficient repeatability in at least 1 intensity map with a range of 12–47 repeatable image features for each map. Assessment of image feature significance with WSR selected for image features from each intensity map based on three criteria: significant change from baseline (scan day 0) on all three subsequent scan days, significant change from baseline on scan day 1 and day 3 or 10, or significant change from baseline on any two scan days. This process of analysis of image feature repeatability and sensitivity to significant change in response to treatment (henceforth, statistical feature selection) produced feature sets for each intensity map later used for multiparametric model development (Fig. 1d).

The average intensity projection (AIP) map contained the most selected image features with 28 showing reproducible data values and significant change from baseline on scan days 1, 3, and 10 compared to dTE image features where 13 met this strictest criterion. Four remaining intensity maps (Baseline, full LIP, LIP, and STD) contained image features with sufficient repeatability and significant baseline changes (ICC ≥ 0.8, p < 0.05 respectively) as previously described.

These image features were then stratified into 3 groups based on the burden incurred by the size of raw datasets for preprocessing, the length of time for image acquisition, and the use of a destructive ultrasound pulse: lowest clinical burden (AIP, LIP, STD) representing 10 frames of raw cine data without contrast destruction, moderate clinical burden (former group plus full LIP) representing all cine frames prior to contrast destruction, and highest clinical burden (former group plus dTE) representing all cine frames before and after contrast destruction (Fig. 1d).

### Features extracted from the AIP intensity map are correlated to the conventional dTE parameter

Taking the difference of AIP and baseline intensity maps yields the dTE map, from which extracted conventional molecular intensity parameters represent the dominant body of data supporting the predictive capacity of 3D-mCEUS. And indeed, previously published investigation^33^ on our raw data demonstrated the dTE mean intensity image feature was 100% accurate in predicting treatment response in tumor models responsive and non-responsive to bevacizumab therapy with a 30% decrease from baseline 24 h after initial treatment. However, the need for a destruction pulse to obtain the dTE limits the feasibility of translating this technique to clinical study; it would thus be ideal if other features not requiring contrast destruction could provide this information.

**Figure 2:**
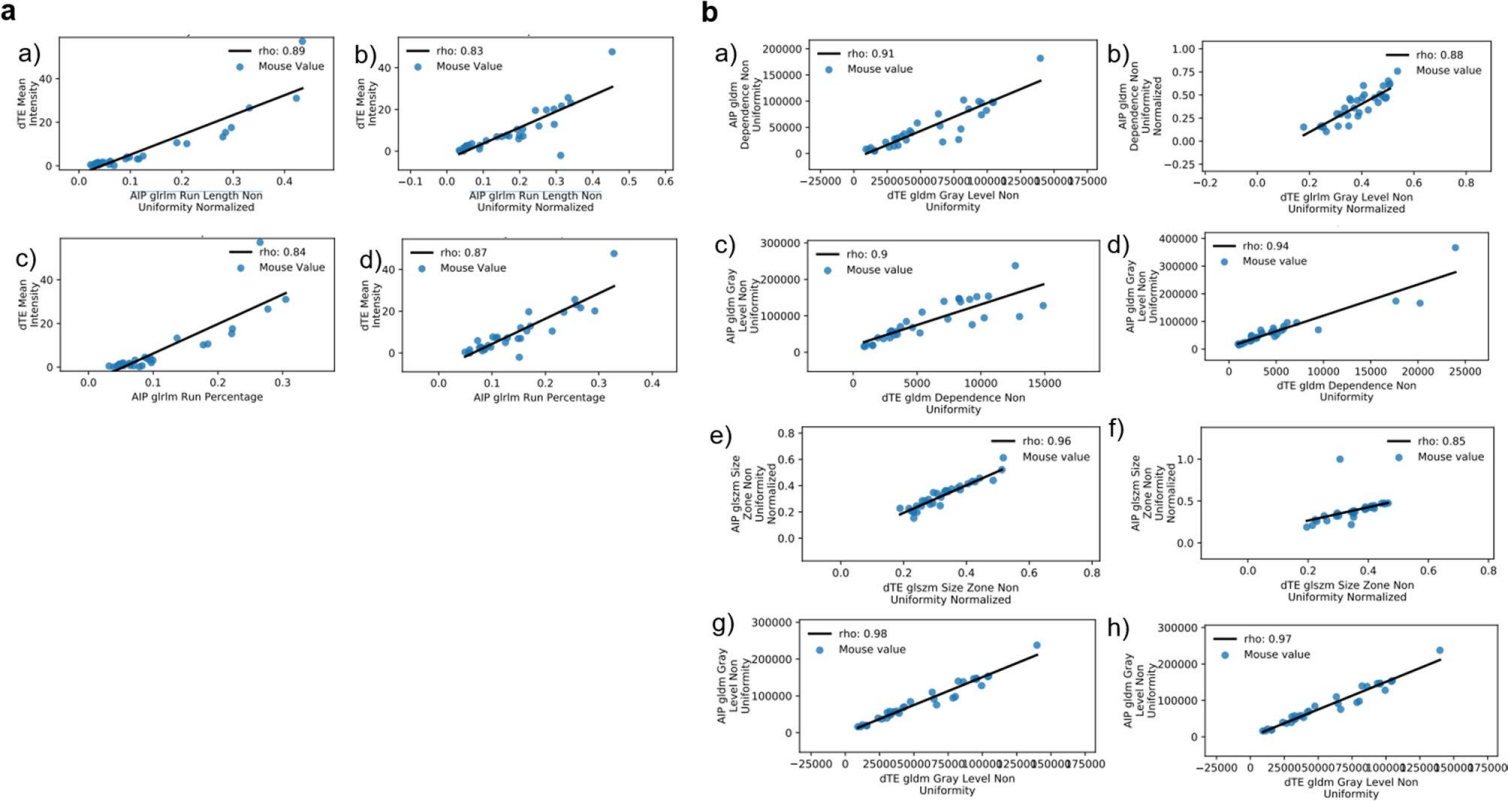
*GLRLM* Gray-level run length matrix, *GLDM* Gray-level dependence matrix, *GLSZM* Gray-level size zone matrix. Bivariate scatter plots of raw feature data comparing selected features and those extracted from spatial mapping of the dTE. (**a**) Scatter plots of raw image feature data showing strong Spearman correlation between the dTE parameter and AIP image features from treated (a, c) and control (b, d) animals. (**b**) Scatter plots of raw image feature data showing strong Spearman correlation between statistically selected features from the spatially mapped dTE parameter and AIP image features in treated (a, c, e, g) and control (b, d, f, h) animals.

Taking the intensity map feature sets from statistical feature selection, we undertook assessment of linear correlation (Spearman correlation; rho > 0.80) comparing the significant and repeatable image features of each intensity map against the dTE mean intensity as well as the 13 dTE image features chosen at statistical feature selection. Only features from the AIP intensity map were found to have linear correlations with dTE image features. Two AIP image features met threshold with linear correlation to the dTE mean intensity (rho ≥ 0.83, Fig. 2a) in both treated and control animals. Spearman correlation assessments between statistically selected features showed strong, linear correlations (rho ≥ 0.85) between several AIP image features and features extracted from the spatial mapping of the dTE parameter in both treated and control animals (Fig. 2b).

### Multiparametric modeling of features from 3D-mCEUS intensity maps is generalizable and predictive of response to anti-angiogenic therapy

**Figure 3:**
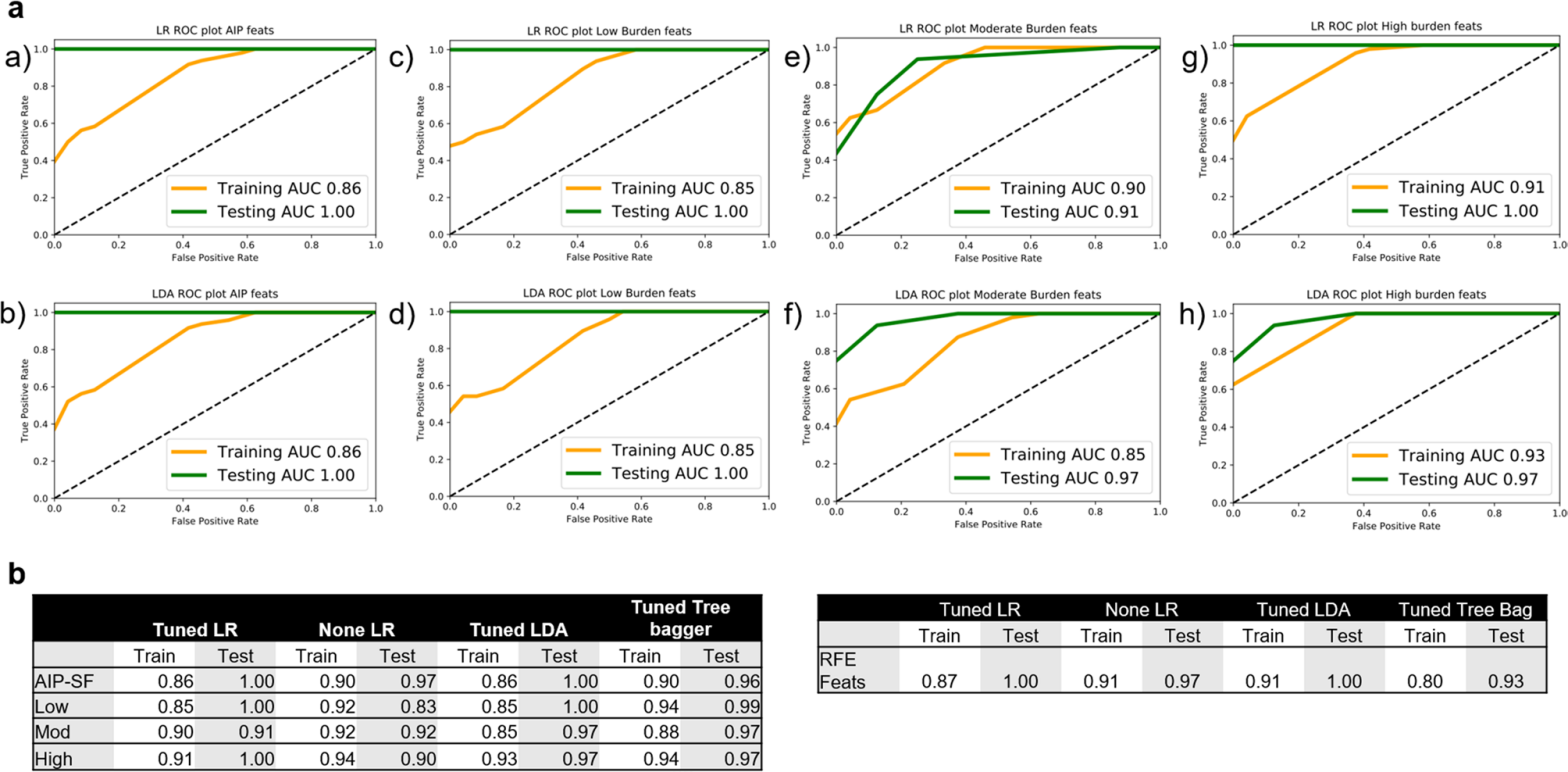
*ROC* receiver operator characteristic, *AUC* Area under the curve, *LR* Logistic regression, *LR None* logistic regression without hyperparameters, *LDA* linear determinant analysis, *AIP-SF* statistically selected average intensity projection image features, *Low* low burden image feature set, *Mod* moderate burden image feature set, *High* high burden image feature set, *RFE feats* image features common to 3 + tested image feature sets after recursive feature elimination. Overview of models’ performance. (**a**) ROC-AUC plots for LR and LDA models of the four tested image feature sets: (a,b) AIP only features; (c,d) Low burden feature set; (e,f) moderate burden feature set; (g,h) high burden feature set. (**b**) Macro-average AUC scores for four tested image feature sets (left table) and dimensionally reduced feature set (right table) after recursive feature elimination.

Following statistical feature selection, extracted image features were grouped based on total amount of raw imaging data used and the use of destruction pulse in generating the intensity maps. One image feature from highly correlated (Spearman rho > 0.80) feature pairs within each set were eliminated. Assessment of classification performance predicting treated versus control animals was carried out with four supervised learning algorithms paired with recursive feature elimination (RFE) as appropriate: logistic regression (LR) with and without hyperparameters, linear determinant analysis (LDA), and an ensemble bagging classifier (BC). Generalizability was optimized using fivefold cross-validation (CV) for hyperparameter tuning, and tenfold CV for RFE. In total, four image feature sets were tested: AIP-only features, low burden group, moderate burden group, and high burden group for a total of 16 simple supervised learning models. Overall, performance on unseen data surpassed training data performance with excellent performance on unseen testing data (Fig. 3a). Notably a reversal of this relationship was observed in the case of LR without hyperparameters in feature sets from low, moderate, and high burden groups (Fig. 3B).

Image features commonly selected at RFE for optimizing performance of LR and LDA models were assembled to produce a dimensionally reduced, less complex feature set comprised of 10 image features (9 from AIP intensity map, 1 from LIP intensity map; henceforth RFE feature set). These were then passed through the same training/testing pipeline described previously, with similar predictive performance (Fig. 3b). The resultant classification rule is available in the supplementary materials under Supplementary Eq. (1).

### Image features selected at RFE are strongly correlated to the dTE parameter and show significant separation of values between treated and control animals

**Figure 4:**
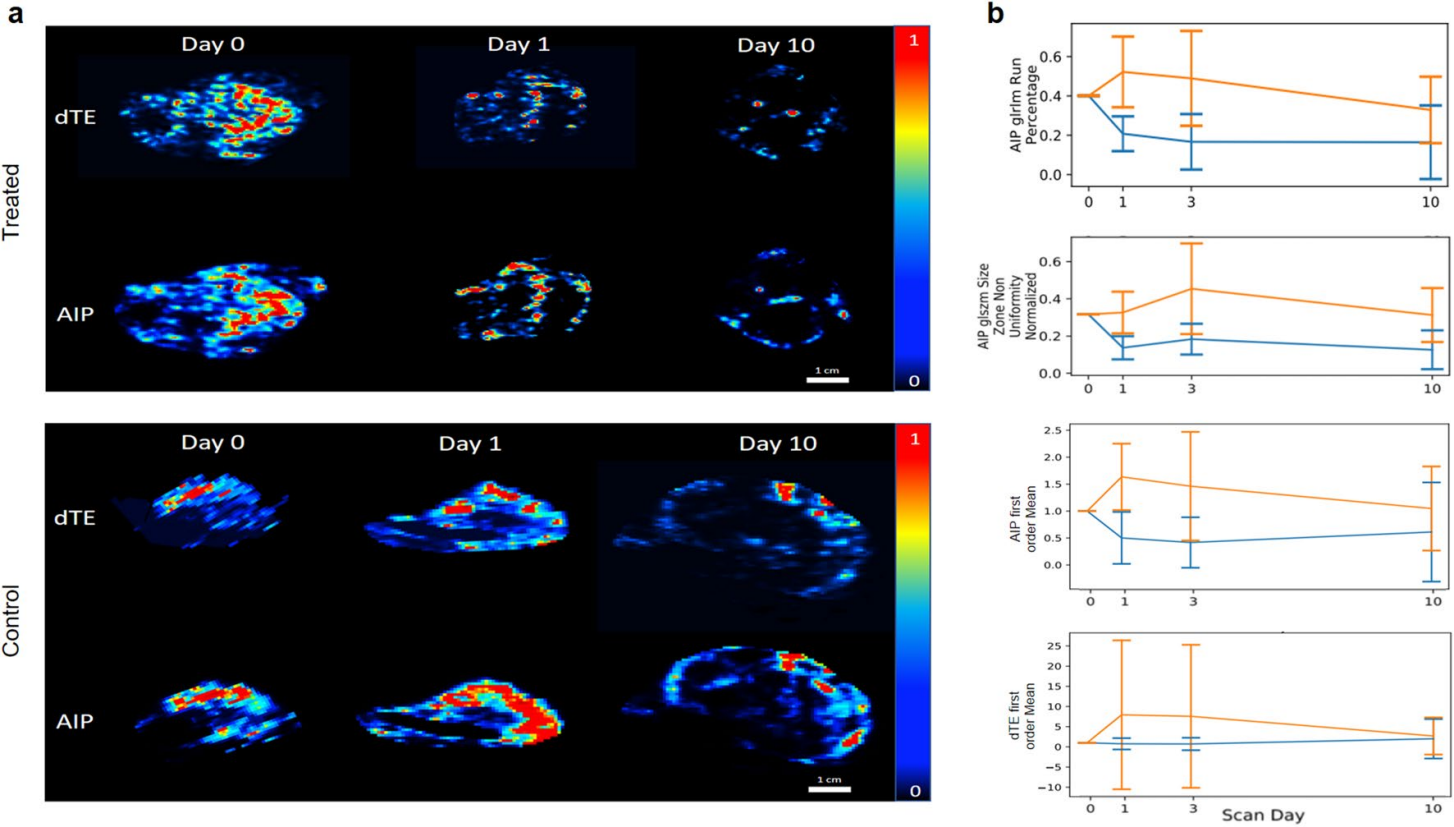
(**a**) Representative image data taken from a single transverse slice through the tumor volume of the AIP and dTE intensity maps of treated and control mice at scan days zero, one, and ten. Images are normalized for gray-scale to be 0–1 and displayed on a color map to allow visual comparison. (**b**) Time-series line plots of average per-animal image feature values with standard deviation error bar for treated animals (orange line) and control animals (blue lines). AIP image features selected at RFE. First order mean feature for AIP intensity map and dTE derived intensity maps showing less resolution of error bar compared to higher-order textural features.

The dimensionally reduced RFE feature set included 9 image features exclusively from low burden group intensity maps (AIP and LIP), which represent data from the last 10 frames of cine image data and do not require destruction pulse. One of the image features composing this set was found to correlate strongly with the conventional dTE parameter (Fig. 2a(c,d)) and three with features extracted from the spatial mapping of the dTE parameter (Fig. 2b(a–f)). Two of these features showed resolution of scan day 1 standard deviation between treated and control animals in line plots of averaged image feature values across scan days, an improvement over previously reported results for mean intensity of the dTE parameter. Figure 4 shows representative image data from which these parameters are derived and line plots of image feature data. A complete list of image features highly correlated with the conventional dTE parameter and the spatial mapping of the conventional dTE parameter can be found in Supplementary Tables 3 and 4, respectively.

## Discussion

Our results demonstrate that the AIP and LIP intensity maps produce a robust collection of reproducible image features, some of which were found sensitive to early molecular changes in treated tumors. When combined in multiparametric analysis, we observed strong performance classifying treated and control tumors equivalent to, or slightly better than, models containing post-destruction data indicating functional molecular imaging for monitoring anti-angiogenic therapy can be reliably carried out utilizing smaller data sets without contrast destruction. Further, several of the AIP intensity map’s image features showed strong linear correlations with the conventional dTE parameter and several image features extracted from the spatial mapping of the dTE parameter. This suggests the mutual encoding of valuable information to these AIP image features for the detection of early response to anti-angiogenic therapy.

The role of VEGFR2 is known to be critically important in mediating angiogenesis in tumors^47,48^, making it a common target for anti-angiogenic therapies such as bevacizumab. And indeed, prior work has shown VEGFR2 overexpression linked with tumor progression and poor prognosis.^49^ Additionally, although heterogeneous expression of endovascular VEGF receptors has been demonstrated in human primary colorectal carcinoma (n = 85), the predominant endothelial receptor was found to be VEGFR2^50^. However, there are currently no clinical tools to assess VEGFR2 expression to understand the significance of these findings and ultimately to support patient management, including therapy monitoring.

Molecular contrast ultrasound as a tool to prospectively detect predictive, quantitative biomarkers of responsiveness of tumor tissue to anti-angiogenic therapy has received much interest in preclinical study and pilot human data with promising results and safety, respectively^20,32,51–56^. This is because it can be used to exclusively image molecular targets on endothelial cells, ideal for characterizing angiogenesis markers such as VEGFR2. An exact understanding of how these derived quantitative parameters relate to the biologic phenomena of treatment response, disease progression, etc. is still unknown. However, advancing non-invasive technologies such as those employed in this work to clinical study would allow for larger study designs to adequately capture the biologic variability seen in clinical cancers. Clinical translation of the traditional disruption approach (i.e. dTE) commonly used with mCEUS has been criticized^57^, and will likely be hampered by concerns of off-target bioeffects of contrast destruction on insonated tissues^58^. Additionally, prior work has focused on extracted intensity parameters averaged over an entire region or volume of interest. Such features are poorly suited to capture spatial heterogeneities characteristic of tumor tissues. The high variance measured in analysis of these parameters are likely in part reflective of variability introduced by inconsistencies in contrast administration, ultrasound probe handling, and differences in manufacturing of microbubble contrast^59^. To overcome these issues, we employed higher-order statistical texture features that provide quantitative parameters of relative differences of intensity values from smaller, sub-ROI neighborhoods of spatially related voxels^38,60,61^. By calculating texture feature values based on the relative intensity of spatially related voxels, we expect resultant parameters to be less susceptible to the extrinsic inconsistencies of imaging acquisition and contrast administration. Interestingly, we observed the range of values from texture features selected in our analysis to be decreased compared to averaged parameters previously reported from these tumors.

Prior work studying similar image texture features in sonographic imaging of cancers have shown promising performance and image feature reproducibility in tumor type discrimination, prognostication, and monitoring of response to therapy^45,62–66^. Application of radiomics analysis to molecular contrast ultrasound data is novel with this being the first study of its kind to the best of our knowledge. In addition, by alleviating the need for contrast destruction to generate predictive models, we see the imminent potential for translation of these methods to the clinical study of wild-type tumor biology. Beyond predicting binary response to anti-angiogenic therapy in clinical settings, the possibility of detecting in vivo the development of acquired resistance or worsening malignant phenotypes deserves exploration.

Our study had several limitations. First, our experimental model was limited to a biologic context where the tumor model used is known to be responsive to bevacizumab therapy^67,68^. Previous publication^33^ that utilized the dTE parameter in molecular imaging included comparison with a pre-clinical tumor model known to be non-responsive to bevacizumab therapy and found these tumors showed molecular intensity dynamics similar to untreated, responsive tumors. Whether the spatial patterns of molecular intensity textures in biologically non-responsive tumors would also be similar is presently unknown. Further, the variability of VEGF receptor expression expected in clinical cancers may be poorly represented in this pre-clinical murine model, limiting the generalizability of our results upon clinical translation. Second, our animal cohort was small for this proof-of-concept work testing the possibility of obviating the need for contrast destruction, and the potential of image features in molecular ultrasound. This small number of observations likely contributed to hyperparameter overfitting^69^, where model performance on unseen data is prioritized at the cost of performance on training data. Future work in this area would benefit from a larger cohort of subjects wherein a dedicated validation set and model calibration would be more feasible. Additionally, applying these methods in other discrete contexts of tumor biology and therapy is needed to determine the biologic underpinnings captured by image feature dynamics.

## Materials/methods

### Experimental design

In this study, we undertook an in-depth analysis of image features extracted from intensity maps of 3D mCEUS images with VEGFR2 microbubble contrast in a murine model of metastatic colorectal cancer. Utilizing data taken from 2 groups of mice—one treated with bevacizumab and one receiving sham infusions—we analyzed statistical properties of extracted image features to select for further analysis those which showed sensitivity to detecting early, durable changes in response to therapy, and those which were reproducible from a data values perspective. These statistically selected features were then stratified into multiparametric feature sets (burden groups) based on the computational requirements and/or necessity of a destruction pulse for producing each image feature’s respective intensity map. The performance of multiparametric feature sets’ predictive capacity classifying treated and control animals was then compared using several simple supervised learning algorithms for the prediction of response to anti-angiogenic therapy 24-h after initial treatment. Further analysis of image features for linear correlation between the conventional dTE molecular intensity parameter and statistically selected dTE image features was carried out to quantify complementarity of information encoded to image features extracted without use of contrast destruction.

### Pre-clinical data groups and measurements

All experiments involving animals were approved by the Stanford Institutional Administrative Panel on Laboratory Animal Care (APLAC) and were performed in accordance with the APLC guidelines and regulations. The present report was prepared in compliance with the ARRIVE guideline. Imaging data from prior published work^33^ were obtained for our analysis. In brief, 16 female nude mice were injected with the human colon cancer cell line LS174T and after 10 days growth period, randomized to a treatment group (n = 8) receiving 10 mg/kg intravenous infusions of bevacizumab and a control group (n = 8) receiving sterile saline infusion on days 0, 3, and 7 after the growth period.

3D mCEUS was performed using a clinical ultrasound scanner (EPIQ 7; Philips Healthcare) with a clinical matrix array transducer (X6-1, 6.0–1.0 MHz frequency, 9212 elements Philips). Reduction of near-field zone artifact of the transducer was achieved with a 3-cm custom standoff ultrasound gel placed to the skin of mice during image acquisition. Scans were performed in power modulation contrast imaging mode for all tumors with transducer fixed in a stable position with a clamp. All mice were anesthetized with 2% isoflurane in room air at 2 L/minute during imaging while positioned on a heated support to maintain stable body temperature for the duration of the experiment.

Contrast used was clinical-grade VEGFR2-targeted microbubbles (MB_VEGFR2+_, 5 × 10^7^ microbubbles/100 µL, BR55, Braco Suisse^70,71^) injected at a constant rate within 5 s using an infusion pump (Kent Scientific) through a 27G needle catheter (Vevo Micromarker, VisualSonics) placed in a tail vein. Total scan time for each tumor each day was 5 min: 4 min to allow complete contrast-perfusion of the tumor volume followed by a sequence of 2 high-powered ultrasound pulses (mechanical index 0.72) applied for 2 s to destroy all bound and unbound microbubbles. The remaining minute allowed tumor volumes to fully replenish intravascular contrast and a second measurement of imaging signal was taken to quantify the bound fraction of molecular contrast from before and after destruction pulses. A further detailed account of image acquisition and contrast injection procedures can be found in Supplementary Methods.

#### Generation of intensity maps

One blinded reader analyzed and manually contoured a volume of interest (VOI) for all 3D mCEUS datasets that includes the entire tumor volume in sagittal, longitudinal, and coronal planes. From these raw imaging datasets, a sliding-window method was used to select all imaging data from prior to contrast destruction, only data from the last 10 frames prior to contrast destruction, or data from before and after contrast destruction. A Python pipeline was used to extract bolus-based parameters of molecular intensity used in conventional analysis on a voxel-by-voxel basis within the manually contoured VOI from the full set of raw imaging data, data from the final 10 frames, and those requiring a destruction pulse. These parameters include average intensity projection, maximum intensity projection, lowest intensity projection, standard deviation, post-destruction baseline, post-destruction standard deviation, and dTE. In total, 11 intensity maps were generated.

### 3D-mCEUS image feature extraction and statistical analysis

Image feature extraction of statistical histogram and radiomics features was carried out using custom software developed in the Python coding language relying on PyRadiomics^41^ for extraction and calculation of data values, generating a total of 1,001 image features from 91 image features for each of 11 intensity maps.

Statistical analysis was performed for feature selection using Python libraries SciPy^72^ and Pingouin^73^ on image feature data normalized as fold change from baseline. Intraclass correlation coefficient (ICC, threshold > 0.80) was calculated using a 2-way mixed model^74^ for the 1001 features using a separate database of serial 3D mCEUS cines obtained from LS174T tumors (n = 18) to determine features with reproducible values. Features that scored above threshold for ICC were kept if also meeting statistical significance by Wilcoxon signed-rank test.

Wilcoxon Signed-Rank test was employed to detect significant (p < 0.05) change from baseline in treated animals. Three p-values were generated for each feature representing changes from baseline on scan days 1, 3, and 10. As such, each intensity map’s reproducible features were considered significant based on 3 criteria: all subsequent scan days from baseline are significantly different from baseline, scan days 1 and day 3 or 10 are significantly different from baseline, and any 2 scan days are significantly different from baseline.

### Analysis of feature correlation

Measurement of strong correlation between intensity map image features and dTE features was explored with Spearman correlation (ρ > / = 0.8) comparing the raw data of treated and control animals’ intensity map image features with image feature data from the dTE map in treated and control animals, respectively. The mean intensity feature of dTE images, shown in prior work to carry predictive potential for detecting response to antiangiogenic therapy^33^ was tested against statistically selected intensity map image features for the detection of strong linear correlation.

Further correlation analysis was carried out similarly comparing statistically selected image feature data of each intensity map against the statistically selected features of the dTE map in treated and control animals, respectively using raw data to quantify the redundant encoding of information in image feature data.

### Multiparametric model development and configuration of model parameters and hyperparameters

Features selected through statistical analysis with Wilcoxon signed-rank test and ICC were stratified into groups representative of the total fraction of TIC necessary to include in calculating intensity maps and the necessity of a destruction pulse phase. The AIP intensity map presented a relatively large set of features (28 image features) and so was made a separate group apart from its image features inclusion in burden group feature sets. As such, four groups were considered: AIP-only features, low burden (AIP, LIP, STD), moderate burden (prior maps plus full LIP), and highest burden (prior maps plus dTE). Burden group feature sets then underwent further feature selection with correlation feature elimination again using Spearman correlation with a high threshold (rho ≥ 0.80) and manual inspection of best-fit scatter-line plots to confirm a linear relationship, eliminating one feature from each high-correlation pair to reduce redundancy of information. Each group’s feature set was then preprocessed as fold-change from baseline value (scan day 0) and scaled to range from 0 to 1. Model tuning and testing was carried out in Sci-kit^75^ implementations of 4 supervised-learning classification implementations of logistic regression with and without hyperparameter tuning, linear determinant analysis, and ensemble bagging classifier. Model training was carried out with data from scan days 0, 3, and 10 with model testing performed on data from scan day 1. Hyperparameter tuning was carried out systematically for each algorithm with fivefold cross-validation before calculating ROC-AUC scores. In the cases of LR with hyperparameters and LDA models, RFE was utilized with tenfold cross-validation.

Image features selected at RFE for each feature set were collected to assess for commonly selected features. Any feature found selected in at least 3 times at RFE were included into a new, dimensionally-reduced feature set and again tested in multiparametric analysis as previously described.

## Supplementary Information

Supplementary Information.

## Data Availability

All data and code for this project can be accessed by interested parties. Requests for access to data and code should be sent to the corresponding author. Execution of the scripts requires a Python environment with packages as stated in the Methods section.
