# Supplementary material for: Characterization of spatially mapped volumetric molecular ultrasound signals for predicting response to anti-vascular therapy
Source: Sci Rep. 2023 Jan 30;13:1686. doi: 10.1038/s41598-022-26273-0 (PMC9886917; doi:10.1038/s41598-022-26273-0)
Supplement: Supplementary file 1 — Supplementary Information. [file 41598_2022_26273_MOESM1_ESM.docx]

**TITLE:**

Characterization of Spatially Mapped Volumetric Molecular Ultrasound Signals for Predicting Response to Anti-Vascular Therapy

**ONE SENTENCE SUMMARY:**

3D molecular contrast ultrasound intensity maps yield multi-parametric machine learning models that can longitudinally discriminate between treated and untreated tumors.

**AUTHORS:**

Cody A. Keller, MD^1^, Shaya Zarkesh^1^, Jianhua Zhou, MD^1^, Amelie Lutz, MD^1^, Dimitre Hristov, PhD^3^, Aya Kamaya, MD^1^, Ahmed El Kaffas, PhD^1,2^

**AFFILIATIONS:**

[1] Department of Radiology, Stanford University, Stanford, CA

[2] Department of Radiation Oncology, Stanford University, Stanford, CA

**CORRESPONDING AUTHOR:**

Ahmed El Kaffas, Ph.D.

Department of Radiology

School of Medicine, Stanford University

1201 Welch Road, Rm P097, Stanford, CA 94305

P: 650-353-6495; Fax: 650-723-1909

Email: elkaffas@stanford.edu

**SUPPLEMENTARY METHODS**

**Colon Cancer Xenograft Model**

All animal data for our experiments were derived from raw imaging data previously published^1^ for determining the predictive potential of the conventional dTE parameter to predict treatment response to anti-angiogenic therapy. All experimental procedures involving laboratory animals were approved by the Institutional Administrative Panel on Laboratory Animal Care. The human colon cancer cell line LS174T (obtained from ATCC January 2015), which was shown to be sensitive to VEFG pathway blockade therapies such as bevacizumab^2^, was cultivated in Dulbecco’s Modified Eagle Medium (DMEM; Gibco, Grand Island, NY) supplemented with 10% fetal bovine serum (Gibco), penicillin (50 U/mL), and streptomycin (50 µg/mL) at 37°C in a humidified 5% CO_2_ atmosphere. After trypsinization, tumor cells were collected and suspended in 50 µL PBS and 50 µL Matrigel (BD Biosciences, San Jose, CA) then injected subcutaneously on the right lower hind limb of total 34 6-8 weeks old female nude mice (Charles River Laboratories, Wilmington, MA). From these n=34 mice, 16 were used for treated (n=8) and control (n=8) groups, and the remaining 18 used for repeatability assessment. ***Tumor growth*** was allowed for 10 days after cancer cell injections, reaching a maximum diameter of 6-13 mm (mean, 10mm) measured by electronic caliper available on the ultrasound system. Animals subsequently randomized to the treated group were injected subcutaneously with bevacizumab (Avastin, 10mg/kg; Genentech, South San Francisco, CA) on imaging days 0, 3, and 7 while control animals received sterile saline.

**Three-Dimensional mCEUS Data Acquisition**

Three-dimensional mCEUS was performed in all experimental animals on days 0, 1, 3, 7, and 10 using a clinical ultrasound system (EPIQ 7; Philips Healthcare, San Diego, CA) with clinical matrix array transducer (X6-1, 6.0-1.0 MHz frequency, 9212 elements; Philips). A 3-cm customized standoff ultrasound gel was positioned on the skin of the animal to reduce artifact in the near-field zone of the clinical transducer, which was fixed in stable position with a clamp to limit motion artifact. For all tumors, image acquisition was performed in power modulation contrast imaging mode using the following settings: center frequency, 3.2 MHz, mechanical index, 0.09; volume rate, 1 Hz; dynamic range, 52 dB; imaging focus, 5cm. These settings were kept constant during all experiments. All mice were anesthetized with 2% isoflurane in room air (administered at 2 L/minute) during imaging while placed on a heated support to maintain a constant body temperature.

Clinical-grade VEGFR2-targeted microbubbles (MB­_VEGFR2+_, 5x10^7^ microbubbles/100µL, BR55, Braco Suisse^3,4^) were injected at a constant rate through a 27G needle catheter (Vevo Micromarker; VisualSonics; Toronto, Canada) placed in a tail vein. Three-dimensional mCEUS images were recorded in real time using a built-in Digital Navigation Link native to the ultrasound system with custom in-house MevisLab modules written in C++.^5^ Following 4 minutes of dwell time to allow adequate circulation and binding of contrast material in tumors, a sequence of 2 high-powered ultrasound pulses (mechanical index 0.72) were applied for 2 seconds duration to destroy all bound and unbound microbubbles. Sixty seconds following contrast destruction, imaging signal was measured again for calculating differential targeted-enhancement and other post-destruction intensity maps.

**Generation of Intensity Maps**

To generate volumes of interest (VOI), a single blinded reader manually contoured each whole tumor visualized in sagittal, longitudinal, and coronal planes using ITK-Snap. A total of 11 molecular signal intensity maps, detailed in Supplementary Table 1, were generated in 3D and saved as NIFTI files. Python-based software was used to generate intensity projection maps using parallel processing on a high-performance, multi-core processing computing cluster to compute statistical properties of intensity dynamics on a voxel-by-voxel basis during the period of each sliding window.

**Histogram and Texture Feature Extraction**

Histogram and texture features were extracted from each intensity map for each mouse on each scan day using a custom Python pipeline including PyRadiomics version 3.0.1 library for feature value calculations. Histogram features included first order statistical features mean, median, skewness, kurtosis, etc. Second order features extracted are grouped by their use of spatial-relational matrices such as the gray-level co-occurrence matrix and gray-level run length matrix. Supplementary Table 2 lists each feature extracted in our analysis.

Intensity map images were programmatically paired with matching volumetric masks generated at manual tumor contouring prior to extraction with PyRadiomics. Parameter settings for extraction were as follows: bin width: 25, label: 1, interpolator: ‘sitkBSpline’, resampled Pixel Spacing: None, weighting Norm: None, Image types: Original. All feature classes and list of features extracted can be found in Supplementary Table 2.

**Feature Data Analysis**

For the purposes of feature selection and multiparametric analysis, all raw feature data of each animal were represented as fold-change from baseline (Scan Day 0) using the formula X_D0_ / X_D0_, X_D1_/X_D0_, *X*_D3_/*X*_D0,_ *X*_D10_/*X*_D0_ to divide each image feature value by the baseline value measured on scan day 0. Such data was then tested for reproducibility with intraclass correlation coefficient (ICC) using a 2-way mixed model (threshold > 0.80). Reproducibility data was gathered from a separate cohort of n=18 nude female mice similarly xenografted with LS174T tumors, serially imaged with the same imaging protocol, and cine images converted to 11 intensity maps. Resulting data from each intensity map and each image feature were used to calculate ICC values determining reproducibility of each image feature from each intensity map, with those scoring above threshold being kept for further analysis.

Wilcoxon Signed-Rank test was then carried out on fold-change data from the experimental animals to determine which reproducible image features were sensitive to early changes from baseline (p<0.05) in response to therapy on scan day 1, 3, and 10; scan days 1 and 3 or 1 and 10; or any combination of 2 subsequent scan days feature value were significantly different from baseline.

**Multiparametric Model Development**

Image features remaining after feature selection were scaled to range from 0 to 1 for each image feature from each intensity map using the following formula: (*X* – *X*min)/ (*X*max – *X*min). This allowed for the comparison of fold-change values from each image feature from intensity maps regardless of difference in the ranges of values. Scaled data were then analyzed for linear correlation (Spearman; rho ≥ 0.08) to reduce redundancy of feature sets by removing one feature from highly correlated pairs of features.

**SUPPLEMENTARY FIGURES**

Supplementary Figure 1: Example image data for each intensity map on scan day 0. Each image here was normalized to an internal maximum intensity to emphasize differences for visual comparison after application of a color map.


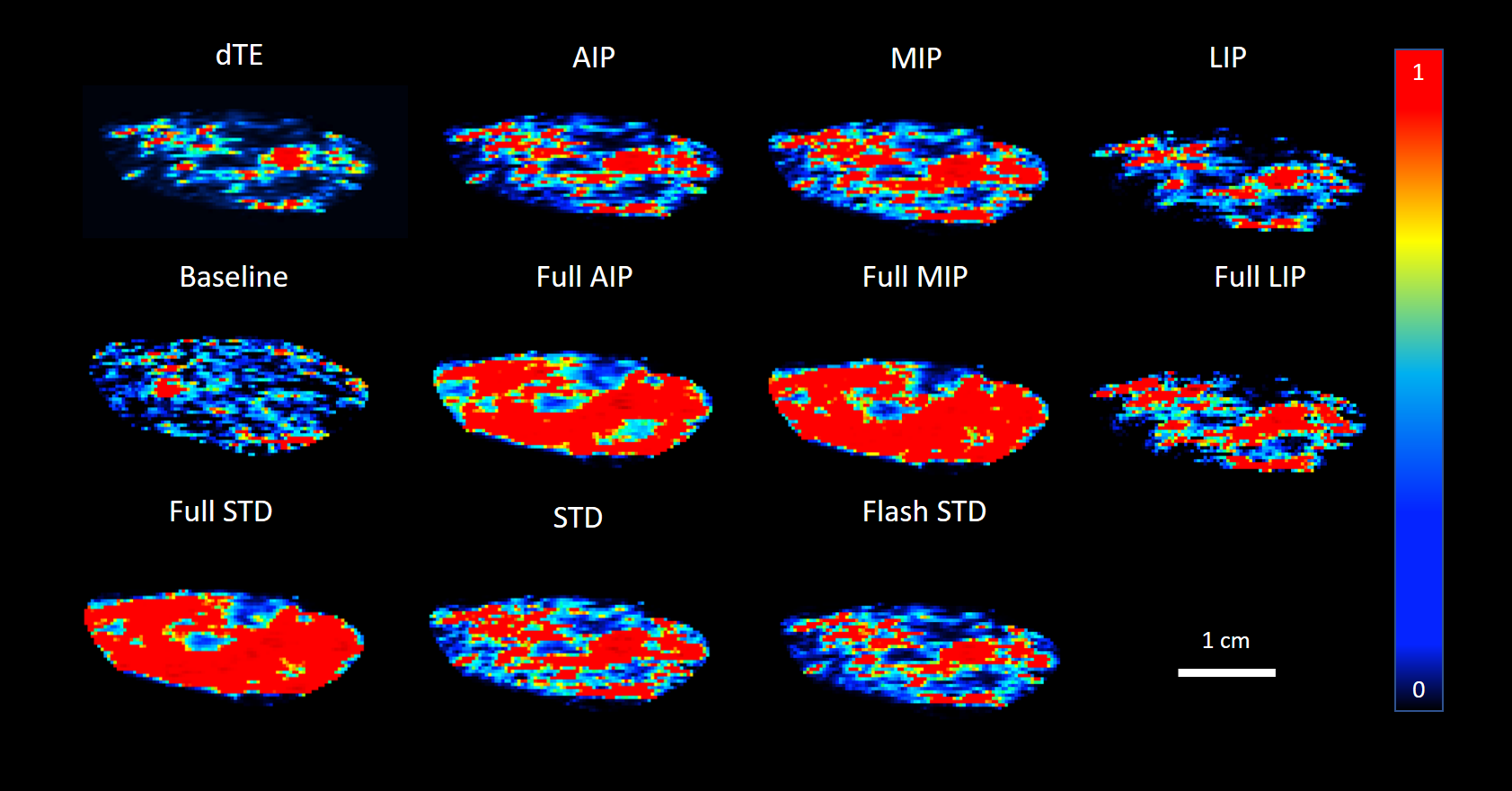


Supplementary Table 1: Detailed explanation of sliding-window method and uniform statistical sampling used to generate the 3D intensity maps. Figure 1B provides a schematic of these windows.

| Intensity Map | Frames extracted | Calculation of new voxel values |
| --- | --- | --- |
| Average intensity projection (AIP) | Final 10 frames prior to destruction pulse | Average intensity value over extracted period |
| Baseline | 60 sec. post-destruction intensity value | Intensity value measured 60 sec. post-destruction |
| Differential targeted enhancement (dTE) | Final 10 frames prior to destruction pulse and 60 sec. post-destruction intensity value | Value derived as the difference of AIP and baseline maps |
| Flash Standard Deviation (FlashSTD) | Final 10 frames prior to destruction pulse and 60 sec. post-destruction intensity value | Standard deviation of intensity values prior and post-destruction |
| Full Average Intensity Projection (FullAIP) | All frames (4 mins.) of wash-in and wash-out prior to destruction pulse | Average intensity value over extracted period |
| Full Maximum Intensity Projection (Full MIP) | All frames (4 mins.) of wash-in and wash-out prior to destruction pulse | Maximum intensity value over extracted period |
| Full Lowest Intensity Projection (FullLIP) | All frames (4 mins.) of wash-in and wash-out prior to destruction pulse | Lowest intensity value over extracted period |
| Full Standard Deviation (FullSTD) | All frames (4 mins.) of wash-in and wash-out prior to destruction pulse | Standard deviation calculated from intensity values over period of extraction |
| Maximum Intensity Projection (MIP) | Final 10 frames prior to destruction pulse | Maximum intensity value over extracted period |
| Lowest Intensity Projection (LIP) | Final 10 frames prior to destruction pulse | Lowest intensity value over extracted period |
| Standard Deviation (STD) | Final 10 frames prior to destruction pulse | Standard deviation calculated from intensity values over period of extraction |

Supplementary Table 2. Summary of extracted features.

| Type | **Features** |
| --- | --- |
| First-order gray level Statistics  Histogram-based features derived from the entire ROI | 10^th^ Percentile  90^th^ Percentile  Energy  Entropy  Interquartile Range  Kurtosis  Maximum  Mean Absolute Deviation  Mean  Median  Minimum  Range  Robust Mean Absolute Deviation  Root Mean Squared  Skewness  Total Energy  Uniformity  Variance |
| Gray Level Co-occurrence Matrix (GLCM) ^6^  Commonly used features employ a co-occurrence matrix assessing for co-occurrence between 2 neighboring pixels using a set direction and scale. | Autocorrelation  Joint Average  Cluster Prominence  Cluster Shade  Cluster Tendency  Contrast  Correlation  Difference Average  Entropy  Difference Variance  Joint Energy  IMC 1  IMC 2  IDM  IDMN  ID  Inverse Variance  Maximum Probability  Sum Entropy  Sum Squares |
| Gray Level Run-Length Matrix (GLRLM) ^6–10^  Matrices based on the run-length of voxels with the same grey level given a specified direction. | Gray Level Non-Uniformity  Gray Level Non-Uniformity Normalized  Gray Level Variance  High Gray Level Run Emphasis  Long Run Emphasis  Long Run High Gray Level Emphasis  Long Run Low Gray Level Emphasis  Low Gray Level Run Emphasis  Run Entropy  Run Length Non-Uniformity  Run Length Non-Uniformity Normalized  Run Percentage  Run Variance  Short Run Emphasis  Short Run High Gray Level Emphasis  Short Run Low Gray Level Emphasis |
| Gray Level Size-Zone Matrix ^6–10^  Using matrices independent of direction, built upon the GLRLM, estimate the size of zones within an image that have the same grey level. | Gray Level Non-Uniformity  Gray Level Non-Uniformity Normalized  Gray Level Variance  High Gray Level Zone Emphasis  Large Area Emphasis  Large Area High Gray Level Emphasis  Large Area Low Gray Level Emphasis  Low Gray Level Zone Emphasis  Size Zone Non-Uniformity  Size Zone Non-Uniformity Normalized  Small Area Emphasis  Small Area High Gray Level Emphasis  Small Area Low Gray Level Emphasis  Zone Entropy  Zone Percentage |
| Gray Level Dependence Matrix ^11–13^  Direction-dependent matrices quantify gray level dependencies of neighboring voxels. | Dependence Entropy  Dependence Non-Uniformity  Dependence Non-Uniformity Normalized  Dependence Variance  Gray Level Variance  High Gray Level Emphasis  Large Dependence Emphasis  Large Dependence High Gray Level Emphasis  Large Dependence Low Gray Level Emphasis  Low Gray Level Emphasis  Small Dependence Emphasis  Small Dependence High Gray Level Emphasis  Small Dependence Low Gray Level Emphasis |
| Neighborhood Gray-Tone Difference Matrix ^14^  Designed to capture grey-scale differences between neighboring pixels, these features have shown comparable texture discrimination to human perception. | Busyness  Coarseness  Complexity  Contrast  Strength |

Supplementary Table 3. Image features extracted from intensity projection maps highly correlated (Spearman rho ≥ 0.80) with the conventional dTE parameter. Only average intensity projection image features were found to have such high correlations. **A** Results from raw feature data for treated animals, **B** raw feature data for control animals.

*Bolded: Feature selected at RFE of burden feature sets | †: Feature selected at LDA RFE of RFE reduced feature set | *: Feature chosen at LR RFE of RFE reduced feature set*

**A**

| Raw dTE Mean int v AIP features; Treated |
| --- |
| firstorder uniformity |
| glcm joint energy |
| glcm Maximum probability |
| **glrlm run length non uniformity normalized** |
| **glrlm run percentage †*** |
| gldm dependence variance |

**B**

| Raw dTE Mean v AIP features; Control |
| --- |
| firstorder uniformity |
| glcm joint energy |
| glcm Maximum probability |
| **glrlm run length non uniformity normalized** |
| **glrlm run percentage †*** |
| gldm dependence variance |
| gldm dependence entropy |
| **gldm dependence non uniformity norm*** |
| gldm large dependence low gray level emphasis |
| **gldm large dependence emphasis** |
| gldm small dependnece low gray level emphasis |

Supplementary Table 4. Image features extracted from intensity projection maps highly correlated (Spearman rho ≥ 0.80) with image features extracted from the spatial mapping of dTE parameter. Only average intensity projection image features were found to have such high correlations. **A** Results from raw feature data for treated animals, **B** raw feature data for control animals.

*Bolded: Feature selected at RFE of burden feature sets | †: Feature selected at LDA RFE of RFE reduced feature set | *: Feature chosen at LR RFE of RFE reduced feature set*

**A**

| Raw dTE map featuress v AIP featuress Treated |
| --- |
| **dTE gldm dependence non uniformity** \| **AIP gldm gray level non uniformity** **†*** |
| **dTE gldm gray level non uniformity** \| **AIP gldm dependence non uniformity** |
| **dTE gldm Gray level non uniformity** \| **AIP gldm gray level non uniformity** **†*** |
| **dTE gldm gray level non uniformity** \| **AIP glrlm run variance *** |
| dTE glrlm gary level non uniformity normalized \| AIP gldm dependence variance |
| dTE glrlm Gray level non uniformity normalized \| AIP firstorder Uniformity |
| dTE glrlm gray level non uniformity normalized \| AIP glcm joint energy |
| dTE glrlm gray level non uniformity normalized \| AIP gldm maximum probability |
| dTE glrlm gray level non uniformity normalized \| AIP gldm small dependence low gray level emphasis |
| dTE glrlm Gray level non uniformity normalized \| AIP glrlm Low gray level run emphasis |
| dTE glrlm gray level non uniformity normalized \| **AIP glrlm run length non uniformity normalized** |
| dTE glszm gray level non uniformity normalized \| Aip glszm low gray level zone emphasis |
| dTE glszm gray level non uniformity normlized \| AIP glszm gray level non uniformity normalized |
| dTE glszm large area emphasis \| **AIP gldm dependence non uniformity** |
| dTE glszm large area emphasis \| **AIP glrlm run variance*** |
| dTE glszm size zone non uniformity normalized \| **AIP glszm size zone non uniformity normalized †*** |
| dTE glszm small area emphasis \| **AIP glszm size zone non uniformity normalized †*** |
| dTE glszm zone variance \| **AIP gldm dependence non uniformity** |
| dTE glszm zone variance \| **AIP glrlm Run variance*** |

**B**

| Raw dTE map features v AIP features Untreated |
| --- |
| **dTE dependence non uniformity** \| **AIP gldm dependence non uniformity** |
| **dTE gldm Dependence Non uniformity** \| **AIP gldm Gray level non uniformity*†** |
| **dTE gldm gray level non uniformity** \| **AIP gldm dependence non uniformity** |
| dTE glrlm Gray level non unifiormity normalized \| AIP gldm Small Dependence Low gray level emphasis |
| dTE glrlm Gray level non uniformity Normalized \| AIP firstorder Uniformity |
| dTE glrlm Gray Level Non uniformity normalized \| AIP glcm Joint Energy |
| dTE glrlm Gray Level Non uniformity normalized \| AIP glcm Maximum probability |
| dTE glrlm gray level non uniformity normalized \| AIP gldm dependence entropy |
| dTE glrlm gray level non uniformity normalized \| **AIP gldm dependence non uniformity normalized*** |
| dTE glrlm Gray level non uniformity normalized \| AIP gldm Dependence Variance |
| dTE glrlm Gray level non uniformity normalized \| **AIP gldm Large dependence emphasis** |
| dTE glrlm Gray level non uniformity normalized \| AIP gldm Large dependence low gray level emphasis |
| dTE glrlm Gray level non uniformity normalized \| **AIP glrlm Run length non uniformity normalized** |
| dTE glrlm Gray level non uniformity normalized \| **AIP glrlm Run percentage*†** |
| dTE glrlm Gray level non unifromity normalized \| **AIP gldm Gray level non uniformity*†** |
| **dTE glrlm run length non uniformity normalized** \| AIP gldm dependence variance |
| **dTE glrlm run length non uniformity normalized** \| **AIP glszm size zone non unifomrity normalized**†* |
| dTE glrlm short run empahsis \| **AIP glrlm run length non uniformity normalized** |
| dTE glrlm short run emphasis \| AIP firstorder uniformity |
| dTE glrlm short run emphasis \| AIP glcm joint energy |
| dTE glrlm short run emphasis \| AIP glcm Maximum probability |
| dTE glrlm short run emphasis \| AIP gldm dependence variance |
| dTE glrlm short run emphasis \| **AIP gldm large dependence emphasis** |
| dTE glrlm short run emphasis \| AIP gldm large dependence low gray level emphasis |
| dTE glrlm short run emphasis \| AIP gldm large dependence low gray level emphasis |
| dTE glrlm short run emphasis \| AIP glrlm gray level non uniformity normalized |
| dTE glrlm short run emphasis \| AIP glrlm low gray level run emphasis |
| dTE glrlm short run emphasis \| **AIP glrlm run percentage*†** |
| dTE glszm Gray Level non uniformity normalized \| AIP glszm Gray level non uniformity normalized |
| dTE glszm Gray Level non uniformity normalized \| AIP glszm Low gray level zone emphasis |
| dTE glszm size zone non uniformity normalized \| AIP glszm gray level non uniformity normalized |
| dTE glszm size zone non uniformity normalized \| AIP glszm low gray level zone emphasis |
| dTE glszm size zone non uniformity normalized \| **AIP glszm size zone non uniformity normalized*†** |
| dTE glszm small area emphasis \| AIP glrlm gray level non uniformity normalized |
| dTE glszm small area emphasis \| AIP glrlm low gray level run emphasis |
| dTE glszm small area emphasis \| AIP glszm gray level non uniformity normalized |
| dTE glszm small area emphasis \| **AIP glszm size zone non uniformity normalized*†** |

Supplementary Equation 1: X1 = LIP GLRLM Short run emphasis, X2 = AIP GLSZM Size zone non-uniformity normalized, X3 = AIP GLDM Dependence non-uniformity, X4 = AIP GLRLM Run length non-uniformity, X5 = AIP GLDM Dependence non-uniformity normalized, X6 = AIP GLDM Grey level non-uniformity, X7 = AIP GLRLM Long run emphasis, X8 = AIP GLRLM Run variance.

*Y = -1.26 + X1(-12.1) + X2(5.69) + X3(4.41) + X4(4.23) + X5(-3.70) + X6(3.69) + X7(-3.16) + X8(-2.06)*

**SUPPLEMENTARY BIBLIOGRAPHY**

1. Zhou, J. *et al.* Vegfr2-targeted three-dimensional ultrasound imaging can predict responses to antiangiogenic therapy in preclinical models of colon cancer. *Cancer Research* **76**, 4081–4089 (2016).

2. Varey, A. *et al.* VEGF 165 b, an antiangiogenic VEGF-A isoform, binds and inhibits bevacizumab treatment in experimental colorectal carcinoma: balance of pro- and antiangiogenic VEGF-A isoforms has implications for therapy. *Br J Cancer* **98**, 1366–1379 (2008).

3. Pillai, R. *et al.* A phospholipid-PEG2000 conjugate of a vascular endothelial growth factor receptor 2 (VEGFR2)-targeting heterodimer peptide for contrast-enhanced ultrasound imaging of angiogenesis. *Bioconjug Chem* **21**, 556–562 (2010).

4. Pysz, M. A. *et al.* Vascular endothelial growth factor receptor type 2-targeted contrast-enhanced US of pancreatic cancer neovasculature in a genetically engineered mouse model: potential for earlier detection. *Radiology* **274**, 790–799 (2015).

5. Schlosser, J., Kirmizibayrak, C., Shamdasani, V., Metz, S. & Hristov, D. Automatic 3D ultrasound calibration for image guided therapy using intramodality image registration. *Phys Med Biol* **58**, 7481–7496 (2013).

6. Haralick, R. M., Shanmugam, K. & Dinstein, I. *Textural Features for Image Classification*. *IEEE TRANSACTIONS ON SYSTEMS, MAN AND CYBERNETICS* vol. 3 (1973).

7. Chu, A., Sehgal, C. M. & Greenleaf, J. F. Use of gray value distribution of run lengths for texture analysis. *Pattern Recognition Letters* **11**, 415–419 (1990).

8. Dasarathy, B. v. & Holder, E. B. Image characterizations based on joint gray level—run length distributions. *Pattern Recognition Letters* **12**, 497–502 (1991).

9. Thibault, G. Indices de forme et de texture : de la 2D vers la 3D : application au classement de noyaux de cellules. (2009).

10. Galloway, M. M. Texture analysis using grey level run lengths. *NASA STI/Recon Technical Report N* **75**, 18555 (1974).

11. Sun, C. & Wee, W. G. Neighboring gray level dependence matrix for texture classification. *Computer Vision, Graphics, and Image Processing* **23**, 341–352 (1983).

12. Wu, C. M. & Chen, Y. C. Statistical feature matrix for texture analysis. *CVGIP: Graphical Models and Image Processing* **54**, 407–419 (1992).

13. Sassi, O. ben, Sellami, L., Slima, M. ben, Chtourou, K. & Hamida, A. ben. Improved Spatial Gray Level Dependence Matrices for Texture Analysis. *International Journal of Computer Science and Information Technology* **4**, 209–219 (2012).

14. Amadasun, M. & King, R. Texural Features Corresponding to Texural Properties. *IEEE Transactions on Systems, Man and Cybernetics* **19**, 1264–1274 (1989).
